# Mutational Analysis of PBRM1 and Significance of PBRM1 Mutation in Anti-PD-1 Immunotherapy of Clear Cell Renal Cell Carcinoma

**DOI:** 10.3389/fonc.2021.712765

**Published:** 2021-08-10

**Authors:** Abudureyimujiang Aili, Jie Wen, Lixiang Xue, Junjie Wang

**Affiliations:** ^1^Department of Radiation Oncology, Peking University Third Hospital, Beijing, China; ^2^Institute of Medical Innovation and Research, Peking University Third Hospital, Beijing, China

**Keywords:** PBRM1 mutation, PBRM1 expression, clear cell renal cell carcinoma, anti-PD-1 immunotherapy, CD4 T cell

## Abstract

Renal cell carcinoma is a common solid tumor. PBRM1 is one of the most mutation-prone genes in clear cell renal cell carcinoma (ccRCC) with the occurrence of mutation in 40% of ccRCC patients. Mutations in PBRM1 have been correlated with the efficacy of immunotherapy. However, the mutation types of PBRM1 are not well characterized. The effects of PBRM1 expression levels in the tumor microenvironment are not well studied. In addition, the mechanism and effect of anti-PD-1 immunotherapy in ccRCC tumor microenvironments are not well clarified. In this study, using bioinformatics methods we analyzed the alternation frequency and expression levels of PBRM1 in various tumors. Next, we experimentally validated their expression levels in ccRCC tissues from human and mouse models. We attempted to clarify the mechanisms of anti-PD-1 immunotherapy in ccRCC with various PBRM1 expression levels. Our results showed that deficiency of PBRM1 protein is correlated with CD4 T cell reduction in human and mouse ccRCC tissues. We also showed that anti-PD-1 Immunotherapy can increase the infiltration of T cells in both PBRM1 high and PBRM1 low tumors but to different degrees. Our study indicates that the reduction of CD4 cells in tumor tissues with low expression of PBRM1 may explain the compromised efficacy of anti-PD-1 immunotherapy in patients with PBRM1 mutated ccRCC. Our study sheds light on the potential of PBRM1 as a therapeutic target in ccRCC.

## Introduction

Renal cell carcinoma(RCC) is a common solid tumor (1) and 75% of RCC is clear cell renal cell carcinoma (ccRCC) (2). Most ccRCC have mutations in genes including VHL, PBRM1, SETD2, etc. (1, 3). Among them, the mutation rate of PBRM1 in ccRCC is about 40% (4). It has been reported that PBRM1 gene encodes the BAF180 protein which is required for the stability of the SWI/SNF chromatin remodeling complex SWI/SNF-B (PBAF) (5). The importance of a gene depends on its function. Functional studies showed that PBRM1 mutation caused genomic instability (6, 7). PBRM1 knockdown has been shown to result in a significant increase in proliferation of RCC cells (8) and loss of PBRM1 contributes to tumor grade in mice (9). It is worth mentioning that VHL mutation is one of the key factors causing ccRCC (10) and PBRM1 restrains VHL loss-driven ccRCC but mutation of the PBRM1 gene alone does not cause ccRCC (11).

Mutation in PBRM1 has been shown to correlate with the clinical benefit of anti-PD-1 therapy (12, 13). However, the characteristics of PBRM1 mutation types are not well studied. Besides, the mechanisms of how PBRM1 mutation affects the tumor microenvironment (TME) and immunotherapies are unclear. It has been reported that the absence or mutation of some genes in tumors may affect tumor-infiltrating T cells (14). The numbers and function of tumor-infiltrating effector T cells are strongly associated with the efficacy of immunotherapy (15). Generally, the higher the number of immune infiltrating effector T cells, the more effective the treatment with immune checkpoint inhibitors (15). Therefore, it is necessary to explore the alternations that occur in PBRM1 and the changes that mutations of PBRM1 cause in tumors. This facilitates the discovery of the features of PBRM1 and it is beneficial to explore its therapeutic targets.

In this study, we analyzed the potential alternations of PBRM1 gene in various tumors using bioinformatics methods and tools. The results showed that PBRM1 is susceptible to mutations in various tumors including ccRCC. We tested its expression levels in different tumors and analyzed the effects on the tumor stages and survival rates. Our results showed that PBRM1 expression is significantly lower in stage IV tumors compared to stage I tumors of ccRCC. We found that mutation, deep deletion and amplification are the three most common types of alternation in the PBRM1 gene. We clarified its mutation types in ccRCC and analyzed the protein-protein interaction network of PBRM1. In addition, we found that mutation or deficiency of PBRM1 protein correlates with CD4 T cell reduction in human and mouse ccRCC tissues. Anti-PD-1 Immunotherapy increase the infiltration of T cells in both high PBRM1 and low PBRM1 tumors but with different levels. This may explain the previous findings that PBRM1 deficiency affects anti-PD-1 therapy (12). Based on our experimental results, we hypothesized that PBRM1 may affect the chemokines that attract T cells to the tumor microenvironment. Further exploration results showed that expression levels of CXCL10, CCL12, ICAM1 and other cell migration-related molecules were decreased. This study explored deeper features of PBRM1 in tumors and shed light on the significance and prospects of PBRM1 to be a potential target in ccRCC.

## Materials and Methods

### Mice

Female BALB/c mice 6–8 weeks of age were purchased from Vital River Lab Animal Technology Company (Beijing, China). All mice were housed and bred in the Peking University Health Science Center animal breeding facilities (Beijing, China) under specific pathogen-free (SPF) conditions. All animal experiments were carried out following the National Institutes of Health guide for the care and use of laboratory animals (NIH Publications No. 8023, revised 1978) and were approved by the Peking University Health Science Center ethics committee.

### Tumor Cells

Mouse ccRCC cell line Renca was purchased from ATCC and maintained in RPMI-1640 containing 10% fetal bovine serum, non-essential amino acids (NEAA), and sodium pyruvate according to the ATCC instruction at 37°C with 5% CO_2_. PBRM1 knockdown Renca cells were gained using shRNA lentiviral particles (GIDL019025, GENECHEM). Knockdown of PBRM1 was performed as described previously (16).

### *In Vivo* Murine Experiments

5 × 10^5^ Renca cells were suspended in 100 µl Matrigel Matrix (BD PharMingen) diluted with sterile PBS at 1:1, and subcutaneously injected into the backs of BALB/c mice.

Anti-PD-1 immunotherapy in mice was performed as described (17, 18) with some modifications. In brief, anti-PD-1 antibody (RMP1-14, BioXcell) and control antibody were administered on days 6(200μg), 9(100μg), and 12(100μg) post-tumor injection. Mice were sacrificed for analysis 39 days post-tumor injection. Tumors were measured by caliper every 3days and tumor volume calculated as length x width^2^ x0.52. At the time of sacrifice for analysis mice were euthanized and subsequent cervical dislocation.

### Antibodies and Reagents

For flow cytometry and FACS purification of tumor-infiltrating T cell subsets, the following fluorochrome-conjugated antibodies were used. Anti-CD45, anti-CD3, anti-CD4, anti-CD8, anti-PD-1 were purchased from Biolegend (San Diego, CA). For western-blot anti-PBRM1 (D3F7O, CST) and anti-β-actin (13E5, CST) were used. For multiplex immunohistochemistry assay anti-PBRM1 (D3F7O, CST), anti-CD4 (SP35, Maixin), anti-CD8 (SP16, Maixin) were used.

### Opal Multiplex Immunohistochemistry

The human ccRCC detection protocol was approved by Peking University Third Hospital. Human ccRCC Tissue microarrays (TMAs) were generated by OUTDO Biotech (Shanghai, China). The protocol was performed as described previously (12). In brief, TMAs were multiplex immunohistochemically stained (PBRM1, 690; CD4, 540; CD8, 570) and scanned with the Vectra image scanning system (Caliper Life Sciences). Data were analyzed using inForm Tissue Finder software (Caliper Life Sciences).

### Statistical Analysis

The student’s unpaired t-test was used for comparing two groups. For survival analyses, Kaplan-Meier survival curves were used and compared using the log-rank test. Data were analyzed with GraphPad Prism 8.0.2. *P<0.05; **P<0.01; ***P<0.001; ns, no significance.

## Results

### PBRM1 Is Differentially Expressed in Various Tumors

**Figure 1:**
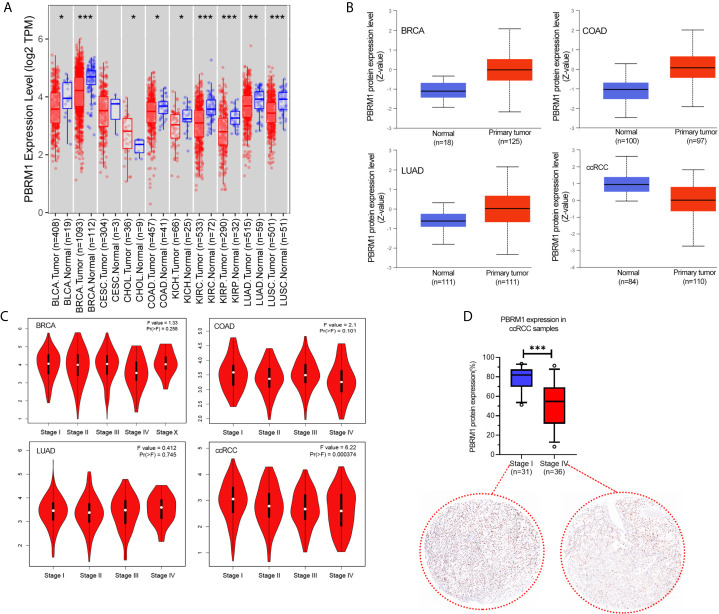
The expression level of PBRM1 varies in different tumors and tumor stages. **(A)** The expression status of the PBRM1 gene in diverse cancer types were analyzed through the TIMER dataset. *P < 0.05; **P < 0.01; ***P < 0.001. **(B)** Based on the CPTAC dataset, the expression level of PBRM1 total protein between normal tissues and primary tumor tissues of breast invasive carcinoma (BRCA), colon adenocarcinoma (COAD), lung adenocarcinoma (LUAD), and kidney renal clear cell carcinoma (KIRC, ccRCC) were analyzed. **(C)** Based on the TCGA data, the expression levels of the PBRM1 gene were analyzed by the main pathological stages (stage I, stage II, stage III, and stage IV) of BRCA, COAD, LUAD, ccRCC. Log2 (TPM+1) was applied for log-scale. **(D)** PBRM1 protein expression levels in Stage I and Stage IV of our ccRCC samples. The expression levels were detected using immunohistochemistry and analyzed by unpaired t-test.

We used the TIMER database to analyze the cancer data in The Cancer Genome Atlas (TCGA) database for PBRM1 expression levels. As shown in **Figure 1A**, in bladder urothelial carcinoma (BLCA), breast invasive carcinoma (BRCA), colon adenocarcinoma (COAD), lung adenocarcinoma (LUAD), kidney renal clear cell carcinoma (KIRC, ccRCC), kidney renal papillary cell carcinoma (KIRP) tumors, there was a significant decrease in the expression level of PBRM1 RNA in cancers compared with normal tissues. Immediately afterward, we used the CPTAC database to validate PBRM1 protein levels in these tumors with differential expression levels (**Figure 1B**). In contrast to the RNA data of TCGA (**Figure 1A**), the expression levels of PBRM1 protein in BRCA, COAD, and LUAD showed a significant difference in the CPTAC database (**Figure 1B**). The results showed that PBRM1 protein level is lower expressed in tumor tissues than in normal tissues only in ccRCC. Next, we analyzed the correlations between tumor grades and PBRM1 expression level (**Figure 1C**). The results showed that the expression level of PBRM1 was lower in COAD in stage IV. In ccRCC, the expression level of PBRM1 showed a decreasing trend with an increasing stage (**Figure 1C**). To further validate, we tested the samples of human ccRCC in Stage I and Stage IV by immunohistochemistry assay in our lab. It showed that PBRM1 was significantly lower in the Stage IV samples (**Figure 1D**). These results suggest that the expression level of PBRM1 varies in different tumors.

### PBRM1 Is Susceptible to Truncating Mutations in ccRCC

**Figure 2:**
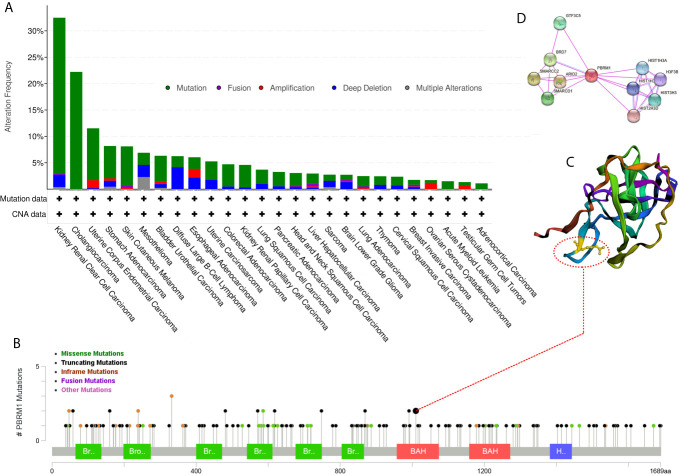
Mutation frequency of PBRM1 in tumors reproduced from the Cancer Genome Atlas (TCGA) database. **(A)** Alternation frequency of PBRM1 in various tumors are shown. **(B)** Mutation types of PBRM1 in ccRCC are shown. **(C)** 3D structure of PBRM1 and the mutation cite is shown. **(D)** Protein-protein interaction network of PBRM1 are shown. The top 10 experimentally determined genes ranked by degree are shown.

To explore the deeper features of alternations of PBR1M, RNA-Seq datasets with corresponding clinical profiles were downloaded from the TCGA database. We used the cBioPortal for Cancer Genomics website (http://www.cbioportal.org) to analyze PBRM1 mutation types in tumors from the TCGA dataset. We found that PBRM1 gene was mutated in a variety of tumors (**Figure 2A**). Interestingly, PBRM1 had the highest probability of mutation in ccRCC (**Figure 2A**). In ccRCC, the most frequent mutation type was truncating mutation (**Figure 2B**). Mutated protein structures may be used to design targeted drugs (19). Thus, we analyzed one of the frequent mutation sites using the cBioPortal website and showed the 3D structure of the site (**Figure 2C**). In addition to considering PBRM1 as a therapeutic target, we can also consider molecules that interact with it as targets. Next, we analyzed the proteins interacting with PBRM1 using STRING (https://string-db.org/cgi/). The top 10 experimentally determined genes ranked by degree were shown (**Figure 2D**). Taken together, these results demonstrated the features of PBRM1 mutations in different tumors and revealed the protein features and protein-protein interaction network of PBRM1. These results revealed the sites of PBRM1 as a potential target.

### PBRM1 Expression Correlates With Tumor Stage and Overall Survival

As it’s shown above, we analyzed the features of PBRM1 at the molecular level. It demonstrates the potential of PBRM1 as a target. But before treating it as a target, we have to further validate its impact in the clinic.

**Figure 3:**
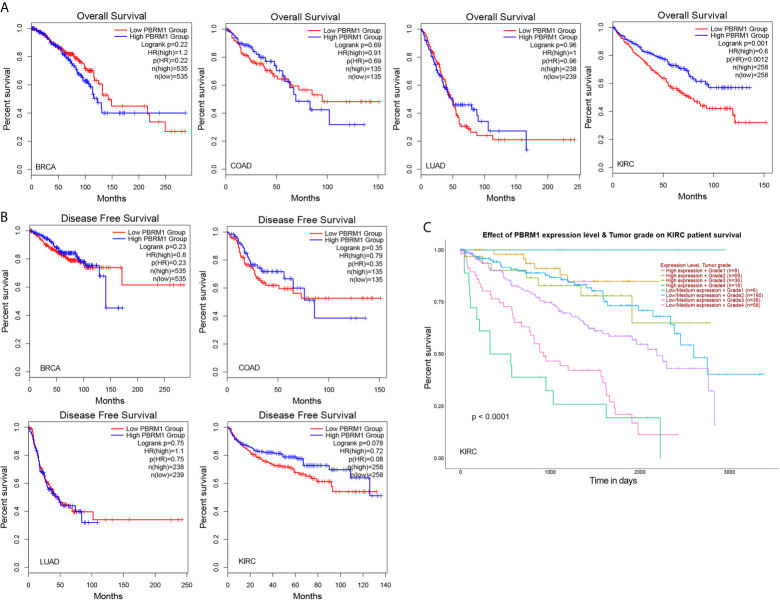
The mutation of PBRM1 affects the survival rates of patients. Overall survival **(A)** and disease-free survival **(B)** of PBRM1 high and PBRM1 low patients in different tumors. GEPIA2 tool was used to analyze the TCGA dataset. BRCA, breast invasive carcinoma; COAD, colon adenocarcinoma; LUAD, lung adenocarcinoma; KIRC, kidney renal clear cell carcinoma (ccRCC). **(C)** Overall survival of KIRC (ccRCC) patients based on the PBRM1 expression level and tumor grade. Kaplan-Meier curves with positive results are given.

Next, using the TCGA database, we analyzed the effect of low PBRM1 expression on the overall survival (OS) of BRCA, COAD, LUAD, and KIRC patients with high or low PBRM1 expression levels. The results showed that PBRM1 expression had little effect on overall survival (OS) in the BRCA, LUAD patients (**Figure 3A**). Meanwhile, in the KIRC patients, the patients with high PBRM1 expression had a significantly higher OS (**Figure 3A**). But in the COAD patients, high PBRM1 expression correlates with lower OS (**Figure 3A**). Then we validated the impact of PBRM1 on disease-free survival (DFS) of those patients. In BRCA and COAD patients, lower PBRM1 expression means longer DFS (**Figure 3B**). No significant differences were found in LUAD. However, in the KIRC, lower PBRM1 expression means lower DFS in 80 and 120 months (**Figure 3B**). Finally, we tested the OS of KIRC patients considering PBRM1 expression and tumor grade at the same time. We found that combination analysis predicts the prognosis of ccRCC patients more accurately (**Figure 3C**). These results suggested that the expression level of PBRM1 had an inconsistent effect on survival in different tumors. In ccRCC, low expression of PBRM1 and high tumor grade imply a worse prognosis. It implies that ccRCC patients may benefit from treatment targeting PBRM1.

### PBRM1 Expression Correlates With CD4 Infiltration in Human ccRCC

**Figure 4:**
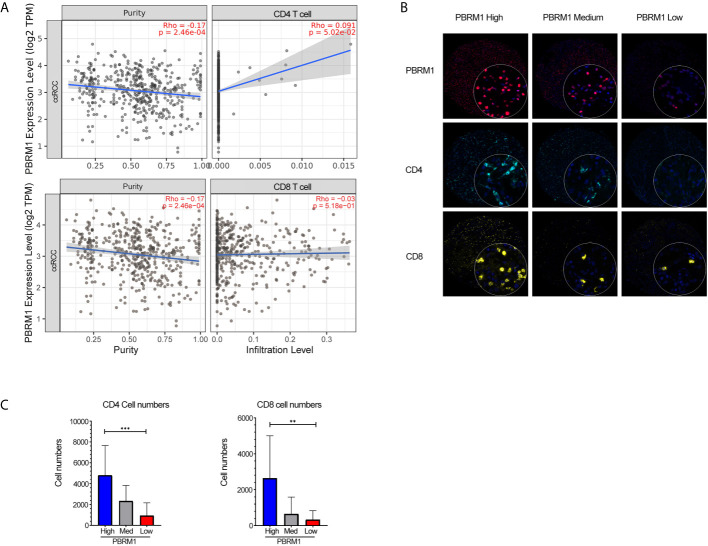
Low expression of PBRM1 correlates with decreased CD4 cells in human ccRCC. **(A)** The correlation of PBRM1 expression with CD4, CD8 within tumors from 533 ccRCC patients were analyzed using the TIMER database. **(B)** Opal Multiplex Immunohistochemistry detection of PBRM1, CD4, CD8 in human ccRCC samples.180 ccRCC samples were tested and classified as High (>80%), Medium (50-60%), Low (<20%) according to the expression level of PBRM1 in the tumor tissues. **(C)** Represents the numbers of CD4, CD8 cells in **(B)**. PBRM1 High, PBRM1 Low groups were calculated by unpaired t-test, **P < 0.01; ***P < 0.001. Data indicates Mean+SD.

To further investigate the influence of PBRM1 mutations in the tumor microenvironment (TME), we performed a correlation analysis of PBRM1 expression levels with CD4 and CD8 cell infiltration using the TIMER database. The analysis showed that CD4 cells positively correlated with the expression levels of PBRM1 in human ccRCC (**Figure 4A**). Meanwhile, the relevance of CD8 cells was not as strong as CD4 cells (**Figure 4A**). To confirm these results, we performed Opal multiplex immunohistochemistry assay of 180 human ccRCC samples. The expression levels of PBRM1 in tumors were classified to High (expression >80%), Medium (expression 50-60%) and Low (expression<20%) groups (**Figure 4B**). We analyzed the number of CD4 and CD8 cells in tumors with different PBRM1 expression levels. We found that in human ccRCC higher level of PBRM1 correlates with more CD4 T cells (**Figure 3C**). Moreover, there was a significant difference in the numbers of CD4 cells between the PBRM1 High group and the PBRM1 Low group (**Figure 4C**). The numbers of CD8 cells also showed a significant difference between the two groups of samples. These results confirmed that PBRM1 mutation affected the tumor microenvironment in human ccRCC.

### PBRM1 Knockdown in Mouse ccRCC Model Mimics PBRM1 Mutation in Human ccRCC

**Figure 5:**
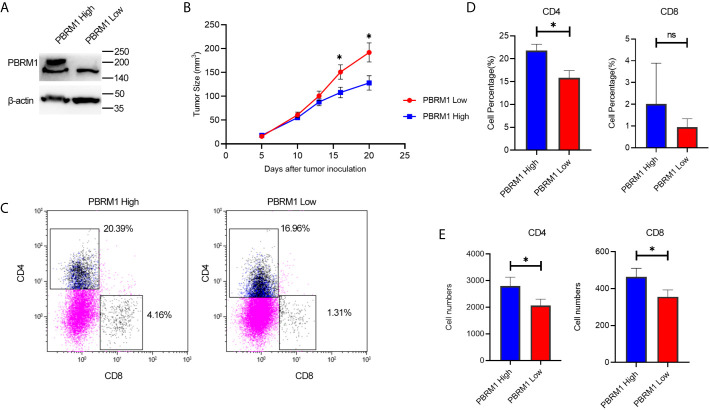
Low expression of PBRM1 causes reduction of tumor-infiltrating CD4 cells in ccRCC mouse model. **(A)** PBRM1 protein expression level in PBRM1 high and PBRM1 low Renca cells were detected by western blot. PBRM1 low Renca cells were obtained using shRNA to PBRM1. β-actin was used as an internal control. **(B)** Growth curves of PBRM1 High and PBRM1 Low tumors in ccRCC mouse model. **(C)** Flow cytometry image of tumor-infiltrating T cells. TIL cells were isolated from tumors and gated CD45^+^ CD3^+^cells. Percentage **(D)** and absolute numbers **(E)** of CD4 and CD8 cells in tumors of PBRM1 High and PBRM1 Low mice model. CD4 andCD8 cells gated in CD45^+^ CD3^+^ cells. Data represents 3 independent experiments at least 6 mice per group. *P < 0.05; ns, no significance.

To explore the effects of PBRM1 mutation on tumor cells and TME, we set up a mouse ccRCC model using the Renca cell line. The Renca is a spontaneous malignancy cell type that originated from the Balb/c mouse (20). RNA (data not shown) and protein level analysis showed that Renca has normal expression level of PBRM1 (Shown as PBRM1 High in the **Figure 5A**). Therefore, we used short hairpin RNA (shRNA) to knock down PBRM1 molecule (PBRM1 Low) (**Figure 5A**). After knocking down PBRM1, we tested the biological characteristics of the cells and observed similar results as previous studies (21). To get the ccRCC mouse model, Renca cells were injected subcapsular. The tumor growth and the survival rate of the mice were observed (**Figure 5B**). After euthanasia, tumor-infiltrating lymphocytes were obtained and counted by flow cytometry for the ratio and number of CD4, CD8 T cells (**Figure 5C**). The results showed that in the PBRM1 Low group, the percentage of CD4 cells was about 15.84%; but in the PBRM1 High group, the percentage of CD4 cells was as high as 21.82% (**Figure 5D**). Moreover, the number of CD8 cells was lower in the PBRM1 Low group. Next, we quantified the absolute number of CD4 and CD8 cells with flow cytometry. We found that the number of CD4 cells was lower in the PBRM1 Low group (**Figure 5E**). Our results concluded that decreased PBRM1 expression levels not only lead to the reduction of CD4 and CD8 cells but also to a decrease in the absolute numbers of CD4, CD8 cells.

These data indicated that, PBRM1 deletion causes a decrease in CD4 cells in the tumor microenvironment of ccRCC.PBRM1 knockdown in mouse ccRCC model mimicked PBRM1 mutation in human ccRCC and this mouse model can be used for further research on the precise mechanisms and therapeutic pathways.

### PBRM1 Expression Level Affects Anti-PD-1 Immunotherapy Efficiency

**Figure 6:**
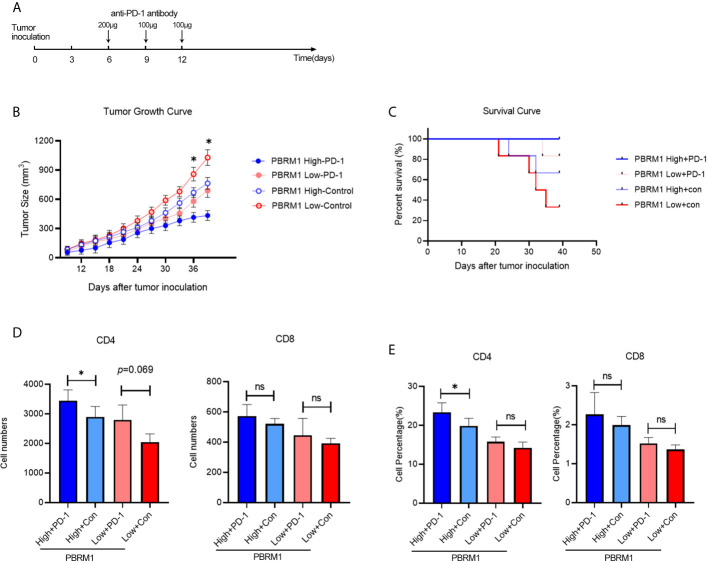
Anti-PD-1 immunotherapy increased CD4 T cell infiltration in ccRCC mouse model. **(A)** Treatment scheme of anti-PD-1 immunotherapy in ccRCC mouse model. Anti-PD-1 antibody and control antibody were administrated at day 6, day 9, and day 12 after tumor inoculation. **(B)** Growth curves of PBRM1 High and PBRM1 Low tumors treated with anti-PD-1 antibody or control isotype antibody in ccRCC mouse model. Test was stopped when mice died or tumors reached 1000mm^3^ in size. **(C)** Survival rates of mice in different groups. Data were analyzed with GraphPad Prism 8.0.2. Absolute cell numbers **(D)** and percentages **(E)** of CD4 and CD8 T cells in tumors of different groups were shown. All live mice were euthanized at day39 and tumor infiltrating T cells were detected by Flow Cytometry. Data are presented as mean ± SD with at least 6 mice for each group. *p < 0.05; ns, no significance.

The findings above suggest that the deficiency of PBRM1 causes immune cell alterations in the TME. It is known that immune cell alterations in the TME of other tumors affect anti-PD-1 immunotherapy (22–24). Thus, we hypothesized that in mouse models, PBRM1 expression levels affects anti-PD-1 immunotherapy. Besides, we wanted to study the mechanisms and effects of anti-PD-1 immunotherapy in ccRCC mouse model. To test our speculation, we performed anti-PD-1 immunotherapy experiments in mice with ccRCC (**Figure 6A**). The results showed that the deletion of PBRM1 affects anti-PD-1 immunotherapy (**Figure 6B**). Tumor growth was significantly inhibited in mice receiving immunotherapy. PBRM1 High mice showed better efficacy in immunotherapy compared to PBRM1 Low mice. Tumors were better suppressed in PBRM1 High mice. Tumor growth was fastest in mice with PBRM1 Low that did not receive PD-1 treatment. Survival curves also showed that anti-PD-1 treatment significantly improved the survival rate of mice (**Figure 6C**).

### Anti-PD-1 Immunotherapy Increased CD4 T Cell Infiltration in ccRCC

We continued to observe the status of the TME after the immunotherapy was completed. To comply with the experimental ethics and animal welfare, we ended the observation when the tumor grew to 1000mm^3^ at day39. Tumor infiltrating T cells were detected with flow cytometry. The results showed that anti-PD-1 immunotherapy increases CD4 T cells in both PBRM1 High and PBRM1 Low groups (**Figures 6D, E**). The deficiency of PBRM1 was associated with a decrease in CD4 and CD8 cells (**Figures 5D, E**). After anti-PD-1 treatment, there was some minor but not significant increase in CD4 and CD8 cells in PBRM1-deficient tumors. However, the PBRM1 High group resulted in a significant increase in CD4 cells after immunotherapy (**Figure 6D**).

The above results showed that the expression level of PBRM1 correlates with the efficiency of anti-PD-1 immunotherapy, and a high expression level of PBRM1 implied a better effectiveness of anti-PD-1 immunotherapy. We found that tumor infiltrating T cells were increased after anti-PD-1 immunotherapy. The increase in the number of infiltrating CD4 and CD8 cells in the TME after anti-PD1 immunotherapy may be one of the mechanisms by which immunotherapy works in ccRCC.

## Discussion

Understanding genetic and epigenetic alterations in cancer can identify new disease mechanisms (25) and new therapeutic targets (26). PBRM1 is one of the most frequently mutated genes in ccRCC (3, 27). But its features of genetic and epigenetic alternations, protein-protein interaction network and the consequences caused by the mutation in the tumors are not well studied. One of the consequences caused by the mutation is that TME alters and may affect the mechanisms of immunotherapy.

To explore the role and commonality of PBRM1 on tumor cells, we analyzed the genetic alternation frequency of PBRM1 in various tumors using bioinformatic methods. The results showed that PBRM1 alters in various tumors. We verified the changes in PBRM1 expression levels in several other tumors, which also showed reduced PBRM1 expression levels in ccRCC, BLCA, BRCA, COAD, LUAD, and other tumors (**Figure 1A**). However, after deeper investigation, we found that there was only a difference at the RNA level, but not at the protein level in most of the tumors (**Figure 1B**). But in ccRCC, both RNA and protein levels were lower in tumor than normal tissue. We validated its expression level in ccRCC tissues derived from human. The results showed that PBRM1 was significantly lower in stage IV tumors than stage I tumors (**Figure 1D**). These results drove us to investigate the features of PBRM1 more deeply. After intensive research, we found that mutation, deep deletion and amplification were the three most common types of alternation in PBRM1 gene (**Figure 2A**). Truncation mutations and missense mutations of PBRM1 are very common in ccRCC (**Figure 2B**). These features may be considered when PBRM1 became a direct target of therapy. In order to validate the signaling pathway of PBRM1, we looked up the protein interaction network of the PBRM1. PBRM1 prefers to interact with DNA-binding or chromatin remodeling proteins such as ARID2, SMARCC2, and BRD7 (**Figure 1D**). These results indicate that mutations in PBRM1 are correlated with genome stability, and alternation in the genomic stability has been considered as a cause of cancer (28, 29). But mutations in PBRM1 alone did not cause ccRCC or other cancers (11).

However, mutations in PBRM1 have showed a negative impact on patient survival in ccRCC and BRCA (30–32).By comparing the effects of PBRM1 on OS and DFS in different tumors, we found that PBRM1 has significantly greater effects on ccRCC than in other tumors (**Figure 3A**). Some investigators have proposed PBRM1 as a target for therapy (33). However, these studies did not clarify the specific mechanism of how PBRM1 affects tumor progression or patient survival. This is partly because previous studies of ccRCC have focused more on the roles of the VHL gene in ccRCC (34, 35). The effects of PBRM1 deletion on tumor cells have been studied in human ccRCC tumor cell lines (36), but these experiments were *in vitro* and detached from the tumor microenvironment *in vivo*. Besides, animal models emerged relatively late (37). Therefore, relatively few studies have investigated the effects of gene mutations in ccRCC on the tumor microenvironment.

In our research, we used the Renca subcutaneous model to investigate the effects of PBRM1 in the ccRCC tumor microenvironment (38). PBRM1 high and PBRM1 low cell lines were obtained and inoculated into the flank of immunocompetent BALB/C mice to create a subcutaneous model. In this regard, the results of our model are closer to the environment *in vivo* compared to *in vitro* experiments. But there is also a disadvantage that this is not a spontaneous tumor model or an orthotopic model. It may not fully mimic the human RCC. However, it is sufficient to study the effect of PBRM1 on cancer cells and the tumor microenvironment (37). Our results show that knockdown of PBRM1 in tumor cells causes the reduction of CD4 T cells in the tumor microenvironment. The numbers of CD4 T cells in the tumor microenvironment are related to the efficacy of PD-1 therapy (39). The efficacy of anti-PD-1 immunotherapy will be better if more CD4 cells are available in TME (40, 41). From this perspective, our results may explain the previous findings that PBRM1 deficiency affects anti-PD-1 therapy (12). Our anti-PD-1 immunotherapy experiments showed that PBRM1 expression have impacts on the immunotherapy. Anti-PD-1 immunotherapy can additionally increase the numbers of CD4 and CD8 T cells in PBRM1 high expressed TME (**Figure 6D**).

Chemokines are one of the factors that are influencing tumor-infiltrating immune cells and tumor immunotherapy in the tumor microenvironment (42, 43). Studies have shown that cancer cells can change the immune landscape by secreting chemokines in ccRCC (44).Based on the above results, we hypothesized that PBRM1 may affect the chemokines that attract T cells to the tumor microenvironment. And further exploration results showed that expression levels of CXCL10 and other cell migration-related molecules were decreased (**Figures S1B, D**). CXCL10 is a chemokine that is involved in attracting CD4 and CD8 cells to the tumor microenvironment in a variety of tumors (43, 45). Based on these facts, it is reasonable to assume that reduction of CXCL10 expression levels in PBRM1 low tumors is one of the factors that is responsible for the reduction of T-cell infiltration. At the same time, we also observed alterations in several other genes and signaling pathways in PBRM1 low tumors (**Figures S1A, C**). Therefore, the exact relationship between the reduction of CD4 cells after PBRM1 deficiency and increase of T cells after anti-PD-1 treatment needs to be further studied.

In conclusion, our work has made three main advances. First of all, we showed RNA and protein levels of PBRM1 in different tumors and the mutation loci of PBRM1 in the ccRCC. This revealed the deeper features of PBRM1 and potentiality of being a therapeutic target in molecular level. Secondly, we analyzed and experimentally proved the variation of PBRM1 protein expression levels within tumors. We also showed that the PBRM1 expression level combined with tumor stage can more accurately reflect the survival rate. This demonstrated the clinical significance of PBRM1 as a target therapy. Thirdly, we have validated the effects of PBRM1 mutations or decreased expression levels on the TME using human and animal samples. We also reported anti-PD-1 immunotherapy of ccRCC causes more T cell infiltration in TME.

Taken together, our data reveals the deeper features of PBRM1 in tumors. PBRM1 mutates in various tumors but it has more significant effect in ccRCC. High PBRM1 expression level and anti-PD-1 immunotherapy imply more T cells in TME. Our study sheds light on the significance of PBRM1 in ccRCC TME and potential of PBRM1 as a therapeutic target in ccRCC.

## Data Availability Statement

RNA-Seq datasets of renal clear cell carcinoma with corresponding clinical profiles was downloaded from The Cancer Genome Atlas (TCGA) database (https://portal.gdc.cancer.gov/). The corresponding information related to patients with the PBRM1 mutation was obtained from the cBioPortal for Cancer Genomics website (http://www.cbioportal.org). The protein-protein interaction was analyzed using STRING dataset. The association between PBRM1 expression and immune infiltrates was analyzed *via* the TIMER (http://timer.cistrome.org/) database. GEPIA2 (http://gepia2.cancer-pku.cn/#index) and UALCAN (http://ualcan.path.uab.edu/index.html) tools were used to analyze TCGA, CPTAC, and GTEx datasets. To analyze the differences between PBRM1 high and PBRM1 low ccRCC cells, we also used the GSE 22316 (human), GSE145919 (mice) data from the GEO database. The data were analyzed using GEO2R, an online analysis tool in the GEO database.

## Ethics Statement

The animal study was reviewed and approved by Peking University Health Science Center ethics committee.

## Author Contributions

AA and LX conceived and designed the study. AA and JW contributed to the experiment and analysis of the data. AA wrote the first draft of manuscript. LX and JJW critically revised the manuscript. All authors contributed to the article and approved the submitted version.

## Funding

This work was supported by the National Natural Science Foundation of China (no.81902909).

## Conflict of Interest

The authors declare that the research was conducted in the absence of any commercial or financial relationships that could be construed as a potential conflict of interest.

## Publisher’s Note

All claims expressed in this article are solely those of the authors and do not necessarily represent those of their affiliated organizations, or those of the publisher, the editors and the reviewers. Any product that may be evaluated in this article, or claim that may be made by its manufacturer, is not guaranteed or endorsed by the publisher.

## Supplementary Material

Supplementary Figure 1 PBRM1 deficiency caused changes in immune cell-associated signaling pathways. **(A)** Top ten significantly up-regulated and down-regulated genes in PBRM1 low ccRCC cells compared to PBRM1 high ccRCC cells. **(B)** Go analysis and KEGG signaling pathway analysis of PBRM1 low ccRCC cells with PBRM1 high ccRCC cells. Significantly changed (*p < 0.05) pathways are shown. **(C)** Significantly changed (*p < 0.05) transcription factor associated genes in PBRM1 low cells compared with PBRM1 high cells. **(D)** Relative mRNA expression of CXCL10 and CCL12 chemokines in PBRM1 high and PBRM1 low Renca cells. Data represents 5 samples per group. *P < 0.05; **P < 0.01; ***P < 0.001.

## Abbreviations

TCGA, The Cancer Genome Atlas; RCC, renal cell carcinoma; ccRCC, clear cell renal cell carcinoma; KIRC, Kidney renal clear cell carcinoma; BRCA, breast invasive carcinoma; COAD, colon adenocarcinoma; LUAD, lung adenocarcinoma; OS, overall survival; DFS, disease free survival; TME, tumor microenvironment.
