# Supplementary figures and images for: Mutational Analysis of PBRM1 and Significance of PBRM1 Mutation in Anti-PD-1 Immunotherapy of Clear Cell Renal Cell Carcinoma
Source: Front Oncol. 2021 Aug 10;11:712765. doi: 10.3389/fonc.2021.712765 (PMC8383204; doi:10.3389/fonc.2021.712765)

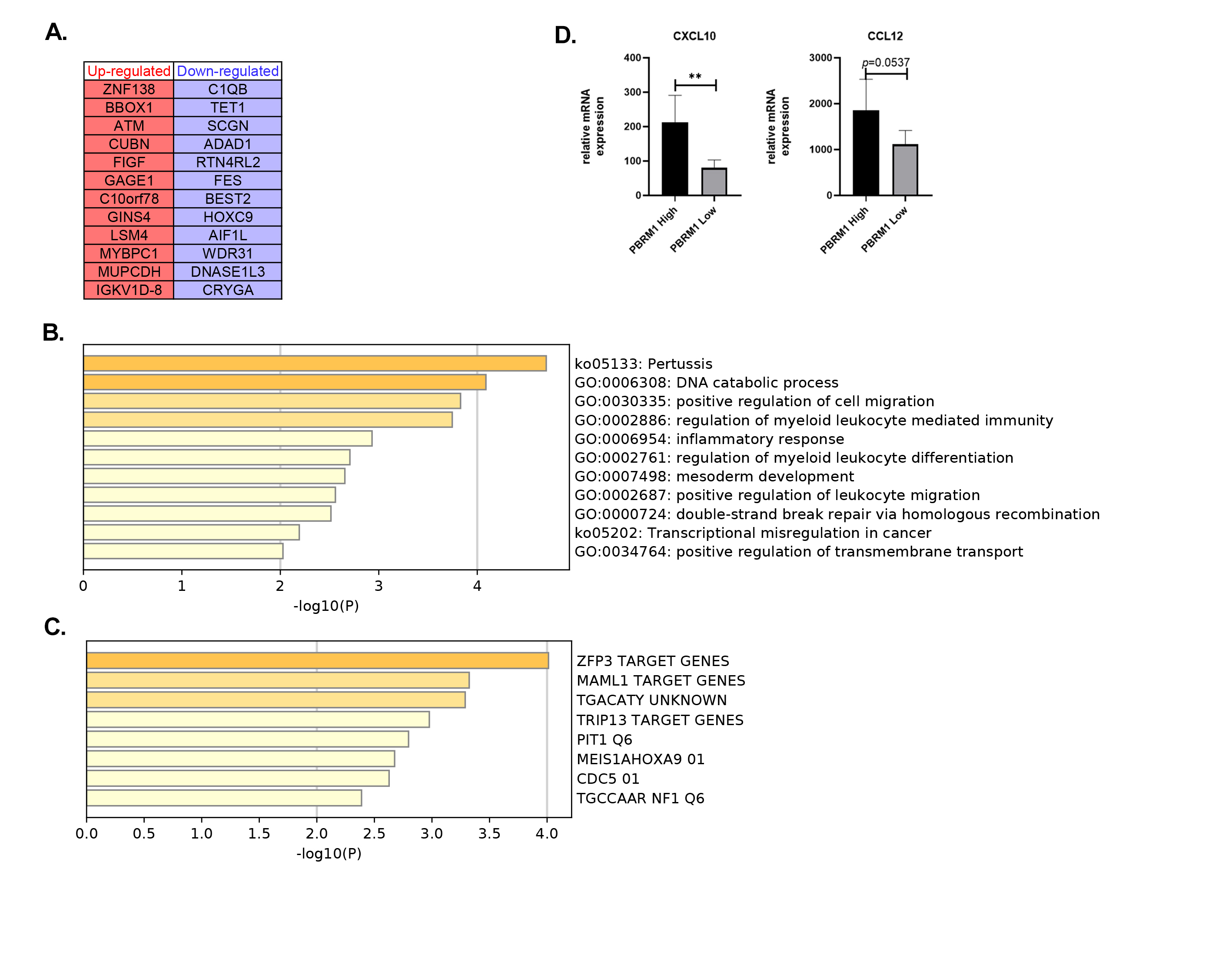

Supplement: Supplementary Figure 1 — PBRM1 deficiency caused changes in immune cell-associated signaling pathways. (A) Top ten significantly up-regulated and down-regulated genes in PBRM1 low ccRCC cells compared to PBRM1 high ccRCC cells. (B) Go analysis and KEGG signaling pathway analysis of PBRM1 low ccRCC cells with PBRM1 high ccRCC cells. Significantly changed (*p < 0.05) pathways are shown. (C) Significantly changed (*p < 0.05) transcription factor associated genes in PBRM1 low cells compared with PBRM1 high cells. (D) Relative mRNA expression of CXCL10 and CCL12 chemokines in PBRM1 high and PBRM1 low Renca cells. Data represents 5 samples per group. *P < 0.05; **P < 0.01; ***P < 0.001. [file Image_1.jpeg]
